# HURP Expression-Assisted Risk Scores Identify Prognosis Distinguishable Subgroups in Early Stage Liver Cancer

**DOI:** 10.1371/journal.pone.0026323

**Published:** 2011-10-17

**Authors:** Ming-Ling Chang, Shi-Ming Lin, Chau-Ting Yeh

**Affiliations:** 1 Liver Research Center and Department of Hepatology, Chang Gung Memorial Hospital, Taoyuan, Taiwan; 2 Graduate Institute of Clinical Medical Sciences, Chang Gung University, College of Medicine, Taoyuan, Taiwan; The University of Arizona, United States of America

## Abstract

**Background:**

Hepatoma up-regulated protein (HURP) is a component of the chromatin-dependent pathway for spindle assembly. We examined the prognostic predictive value of HURP in human hepatocellular carcinoma (HCC).

**Methods:**

HURP expression was evaluated by immunocytochemistry of fine needle aspirated hepatoma cells in 97 HCC patients with Barcelona Clinic Liver Cancer (BCLC) stage A. Subsequently, these patients underwent partial hepatectomy (n = 18) or radiofrequency ablation (n = 79) and were followed for 2 to 35 months. The clinicopathological parameters were submitted for survival analysis.

**Results:**

HURP expression in aspirated HCC cells was detected in 19.6% patients. Kaplan-Meier survival analysis showed that positive HURP expression (*P* = 0.023), cytological grading ≥3 (*P* = 0.008), AFP ≥35 ng/mL (*P* = 0.039), bilirubin ≥1.3 mg/dL (*P* = 0.010), AST ≥50 U/L (*P* = 0.003) and ALT ≥35 U/L (*P* = 0.005) were all associated with a shorter disease-free survival. A stepwise multivariate Cox proportional hazard model revealed that positive HURP expression (HR, 2.334; 95% CI, 1.165–4.679, *P* = 0.017), AST ≥50 U/L (HR, 3.697; 95% CI, 1.868–7.319, *p*<0.001), cytological grade ≥3 (HR, 4.249; 95% CI, 2.061–8.759, *P*<0.001) and tumor number >1 (HR, 2.633; 95% CI, 1.212–5.722, *P* = 0.014) were independent predictors for disease-free survival. By combining the 4 independent predictors, patients with different risk scores (RS) showed distinguishable disease-free survival (RS≤1 vs. RS = 2, *P* = 0.001; RS = 2 vs. RS = 3, P<0.001). In contrast, the patients cannot be separated into prognosis distinguishable subgroups by using AJCC/UICC TNM staging system.

**Conclusion:**

HCC patients with BCLC stage A can be separated into three prognosis-distinguishable groups by use of a risk score that is based upon HURP expression in aspirated HCC cells, ALT, cytological grade and tumor number.

## Introduction

Using an integrative bioinformatics approach to analyze sequence tags expressed in human liver, a novel cell cycle regulated gene named hepatoma up-regulated protein (HURP) was identified 10 years ago [1]. HURP, expressed abundantly in human hepatocellular carcinoma (HCC, ie. hepatoma), is a mitotic phosphoprotein substrate for Aurora-A [2]. Aurora-A is a cell cycle-regulated serine/threonine kinase that displays peak levels of expression during the G2/M phase [3], [4]. The fact that the levels of HURP fluctuate during the cell cycle and reach a peak at G2/M suggests that it plays a role in cell cycle regulation [5]. Further studies have indicated that HURP is a component of the chromatin-dependent pathway for spindle assembly. It has a crucial role in chromatin-induced microtubule assembly, stabilizes and bundles K-fibers, and is essential for de novo microtubule production from chromosomes [6]. Additionally, its activity is required for proper kinetochore capture, efficient chromosome congression, and timely mitotic progression. Defects in these processes can trigger inappropriate anaphase initiation and genomic instability [7], [8]. Aside from transcriptional regulation, intracellular abundance of HURP is also regulated by Cdk1/cyclin B at the posttranslational level [9], [10]. However, there may be some redundant pathways compensating for the function of HURP in the cell cycle as HURP (−/−) mice develop normally and are indistinguishable from their wild-type littermates. The only documented phenotype for HURP (−/−) mice is that female mice are unable to form implantation sites due to an inability to undergo the decidual reaction [11].

Despite the experimental data indicating a link between cell cycle dysregulation and HURP aberrance, no convincing evidence has been established to date suggesting a direct oncogenic role of HURP in HCC. However, pieces of evidence implicating an oncogenic potential of HURP were sporadically reported. Positive HURP expression was associated with the emergence and recurrence of transitional cell carcinoma [12], [13]; gene expression analysis revealed that HURP represented a prognosis marker capable of distinguishing between benign and malignant adrenocortical tumors [14], [15]; and in 293T cell lines (American Type Culture Collection (ATCC) Manassas, VA, USA), overexpression of HURP in differentiated cells increased cell growth and blocked apoptosis that is normally induced by serum starvation [16]. On the other hand, the HURP gene is capable of enhancing the chemosensitivity of deoxycytosine analogs in NIH3T3 cells [17], and the viral protein HBx activates the expression of HURP to prevent apoptosis during cancer progression and establishment of chemoresistance in Hep3B cells [18].

HCC accounts for 90% of primary liver neoplasms, represents the fifth most common cancer in the world, and is the third leading cause of cancer-related death worldwide [19], [20]. A precise staging of the disease may help clinicians to understand the prognosis and make the right choice of therapeutic modalities to benefit patients. Currently, there are several prognostic scoring systems that have been established using different clinicopathological variables [21]. However, even between patients at the same stage of HCC and categorized by the same scoring system, the post-therapeutic prognosis is still diverse. This is most likely due to the fact that HCC is a multi-etiological disease with complex underlying pathogenic mechanisms caused by a variety of risk factors. Presumably, inclusion of good molecular markers in a prognostic prediction system may remedy these insufficiencies and improve the current staging methods [22]. Owing to the availability of ultrasound examination as well as other sophisticated imaging methods, an increasing number of HCCs are detected at an early stage. Furthermore, to minimize the invasiveness of the procedures, pathological diagnosis is gradually replaced by cytology through fine needle aspiration. In addition, surgical resection is being replaced largely by radiofrequency ablation (RFA) because of the comparable therapeutic effectiveness between the two treatments. Cytological characteristics of HCC cells, including differentiation grading and immunostaining of specific antigens, are easily obtained from fine needle aspiration. These parameters are currently not included in any of the scoring systems, but they may provide important information for effective prognosis prediction. Though HURP was first mined from the database of human HCC up-regulated genes, its role in human HCC *in vivo* has remained elusive. To address this, we have established an immunohistochemistry staining method to detect HURP expression in aspirated HCC cells from patients. The clinicopathologic features, cytological grading and HURP expression in HCC cells were all taken into account to calculate the prognostic predictors in these HCC patients.

## Materials and Methods

### Patients

This was a single center, prospective prognostic study that was conducted after approval by the Institutional Review Board at Chang Gung Medical Center. Written informed consent was obtained from all participants before inclusion. From November 2007 through December 2009, 97 consecutive patients (62 males and 35 females), who were diagnosed to have HCC by aspiration cytology and at least two dynamic imaging studies (dynamic computed tomography and angiography), were included in the study. These patients either met the criteria for RFA treatment [23] or had localized HCCs and were suitable for surgical removal of tumors. Blood biochemistries for the following parameters were assayed: aspartate aminotransaminase (AST, <34 U/L), alanine aminotransaminase (ALT, <36 U/L), total bilirubin (Bil, <1.3 mg/dL), alpha-fetoprotein (AFP, <15 ng/mL), albumin (3.5–5.5 g/dL), Prothrombin time (10–13 seconds), creatinine (F:0.44–1.03, M:0.64–1.27 mg/dL). Hepatitis B virus surface antigens (HBsAg) were assayed by a commercially available radioimmunoassay kit (Ausria-II, HBsAg-RIA; Abbott Laboratories, North Chicago, IL). Antibodies to Hepatitis C virus (HCV Ab) were assayed using a third-generation enzyme immunoassay (Ax SYM HCV III, Abbott Laboratories, North Chicago, IL).

Additionally, the following clinicopathological data were also recorded: gender, age, presence of liver cirrhosis, alcohol usage, Edmondson's cytological grade, number of tumors, largest tumor size, presence of ascites upon therapy, date of therapy (RFA or surgery), date of tumor recurrence, and date of last follow-up or HCC related death. In our medical center, patients with main portal vein thrombosis were excluded from surgical or ablation therapy.

### Liver aspiration to diagnose HCC

Under ultrasonographic guidance, a 21- or 22-gauge percutaneous transhepatic cholangiogram needle was used for aspiration cytology. The air-dried smears were immediately stained with Riu's method [24]. Grading of HCC was made by Edmondson and Steiner's classification [25]. If the specimen was insufficient or difficult for cytological diagnosis, an immediate liver biopsy for pathologic examination was undertaken [26].

### HURP immunocytochemistry

Mouse anti-HURP antibodies were kindly provided by Prof. Chou CK (Yang-Ming University, Taiwan). The specificity and sensitivity of these antibodies have been characterized in previous publications [1], [11], [16], [27]. HURP-positive and negative HCC tissues (according to Western blot analysis) were used as controls for each batch of staining. Normal macrophages, lymphocytes, and granulocytes in the cell smears were used as internal negative controls. Aspirated HCC cells were fixed in pure methanol. Hepatocyte expression of HURP was assessed by the avidin-biotin immunoperoxidase method. The slides were incubated in Phosphate buffered saline (PBS) containing 3% hydrogen peroxide for 20 minutes and were subsequently washed twice (5 minutes each) in PBS containing 0.025% Triton X-100 (Sigma Chemical Co., St. Louis, MO). The slides were then incubated with 10% normal horse serum for 30 minutes, followed by an incubation with a 1∶500 dilution of the mouse anti-HURP antibody at 37°C for 1 hour. After being washed with phosphate-buffered saline (PBS; 0.1 M, pH 7.4), the sections were subsequently incubated with biotin-conjugated horse anti-mouse immunoglobulins (Jackson Immunoresearch Lab., West Grove, PA) at a 1∶400 dilution for 40 minutes. After being rinsed with PBS, sections were treated with avidin-biotin complex (Vectastain Elite ABC Kit, Vector Labs, CA) for 30 minutes and then incubated in a diaminobenzidine solution (DAB, Vector Labs, CA) for 1 minute. Nuclear counterstaining was performed with hematoxylin.

### Tumor Ablation

The patients were treated with the internally cooled RF ablation system (Valleylab™, Boulder, Colorado, USA). All RF ablations were performed by three gastroenterologists with ample experience of ablative techniques. The details of tumor ablation were described previously [28].

### Surgical removal of tumor

Tumors were completely resected, with a safety-margin of over 1 cm.

### Follow-up studies

For the patients who received RFA, computed tomography or magnetic resonance imaging was performed 3 weeks later to assess whether the ablation was complete [28], [29]. Following complete ablation or surgical resection, follow-up was performed by ultrasonography, chest X-ray, AFP, and blood biochemistry every 1 to 3 months in the first year and every 3 to 6 months thereafter. Abnormal findings were verified by computed tomography or magnetic resonance imaging. Intrahepatic recurrence was established by the use of the criteria described elsewhere [30]. Depending on the location of the lesions as well as the condition of the patient, extrahepatic recurrence was confirmed by biopsy, aspiration cytology, computed tomography or magnetic resonance imaging [30].

### Statistics

Disease-free survival was measured from the date of diagnosis to the date of recurrence, metastasis, death or last follow-up. The Kaplan-Meier method was used to estimate the survival probability, and the log-rank test was used to compare the survival curves between groups. To determine the cutoffs of a factor with parametric data, experimental univariate analysis was performed to evaluate the association between the factor and disease-free survival using a series of increasing values as the cutoffs. This method was successfully used to identify clinical and virological prognostic factors in HCC patients [30]. The experimental cutoffs were calculated using the following formula: the smallest value+n/15×(the largest value – the smallest value) (n = 1 to 14). As such, a serial of cutoff values were generated for each parametric factor. The experimental dichotomous groups were thus separated by a cutoff at least 1/15 or at most 14/15 of the factor range. This way of grouping was more readily to be used for making treatment recommendations in the future. The cutoff leading to the smallest P value was then selected for subsequent Cox proportional hazard analysis. The justification as well as the limitation of this minimum P-value approach in clinical studies had also been discussed in a review [31]. Stepwise Cox proportional hazard models were used to predict independent predictors associated with disease-free survival. The results are expressed as hazard-rate ratios (HRs) with 95% Confidence interval (CI). In this study, the Bonferroni correction for multiple-comparison was not applied on account of two reasons. First, many of the factors included were known prognostic factors but not randomly selected unknown factors. Our purpose was to understand how significant the HUPR expression was in comparison with these known factors. Second, our final goal was to establish a combination scoring system. Therefore, candidate factors that were possibly significant needed to be included.

Statistical analysis was conducted using SPSS software (version 18.0).

## Results

### Clinical parameters

The baseline characteristics of the 97 patients are listed in table 1. All of them belonged to the Barcelona Clinic Liver Cancer (BCLC) stage A. HBV and HCV infection accounted for the majority of our cases. Almost 90% of the patients were cirrhotic. Most of the patients had abnormal liver function with the mean AST and ALT levels higher than normal limits. However, only a minority of the patients had severe complications (e.g., ascites: 11.3%), while the mean levels of albumin, bilirubin and prothrombin time were within the normal limits. 75.3% of the patients had solitary HCC and only 5.2% of the patients had microvascular invasion. The tumor size ranged from 1.3 to 5.0 cm in diameter. 81.4% of the patients received RFA, whereas the remaining patients had tumors removed surgically. According to the 6^th^ edition of AJCC/UICC TNM Classification, there were 69 and 28 patients belong to Stage I and Stage II respectively.

**Table 1:** Basic clinical parameters for HCC patients.

Clinical parameters	Value
Total number of patients	97
Gender-male, n (%)	62 (63.9%)
Age (years)	65.8±9.8
HBsAg-positive, n (%)	49 (50.5%)
Anti-HCV-positive, n (%)	42 (43.3%)
Alcoholism, n (%)	19 (19.6%)
Cirrhosis, n (%)	87 (89.7%)
RFA^a^, n (%)	79 (81.4%)
Microvascular invasion^b^, n (%)	5 (5.2%)
Ascites, n (%)	11 (11.3%)
Cytology grading <3, n (%)	67 (69.1%)
Solitary tumor, n (%)	73 (75.3%)
Tumor size (diameter, cm)	3.14±2.0
Alpha-fetoprotein (ng/mL)	53.6±104.2
Albumin (g/dL)	3.7±0.5
Bilirubin (mg/dL)	1.1±0.7
Prothrombin time (seconds)	12.8±1.5
Creatinine (mg/dL)	1.4±1.8
AST (U/L)	58.0±48.9
ALT (U/L)	49.1±43.5

a Other patients had tumors removed surgically.

b Post-surgery specimens.

### Expression of HURP in HCCs

**Figure 1:**
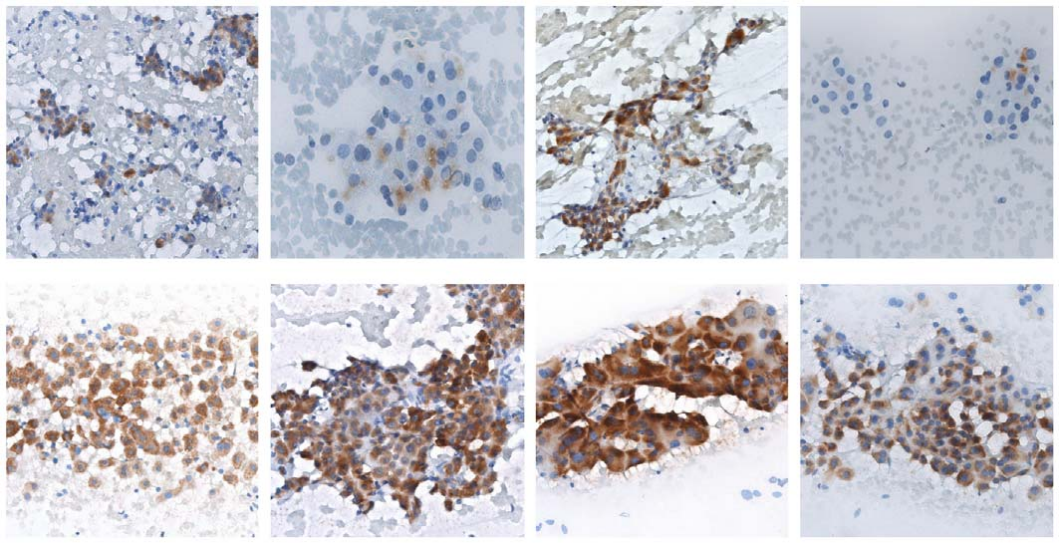
Immunohistochemistry analysis for HURP expression in aspirated human HCC cells in 8 representative cases. HURP was stained in brown color.

Among the 97 patients included, positive expression of HURP was found in the aspirated HCC cells of 19 patients (19.6%). Eight representative cases in which the aspirated cells positively stained with anti-HURP are shown in figure 1. HURP expression was detected in over 80% of the aspirated cells in 15 patients and expression was located in the cytoplasm of the HCC cells (figure 1, lower panel). However, in the remaining 4 patients, <50% of HCC cells were positively stained (figure 1, upper panel). In 2 of these 4 patients, only a few scattered HURP positive HCC cells were found.

To understand whether HURP expression was associated with any of the clinicopathological parameters, logistic regression analysis was performed. It was found that HURP expression was not significantly associated with any clinicopathological parameter (P>0.05 for all clinicopathological factors).

### Association between clinical parameters and disease-free survival

**Figure 2:**
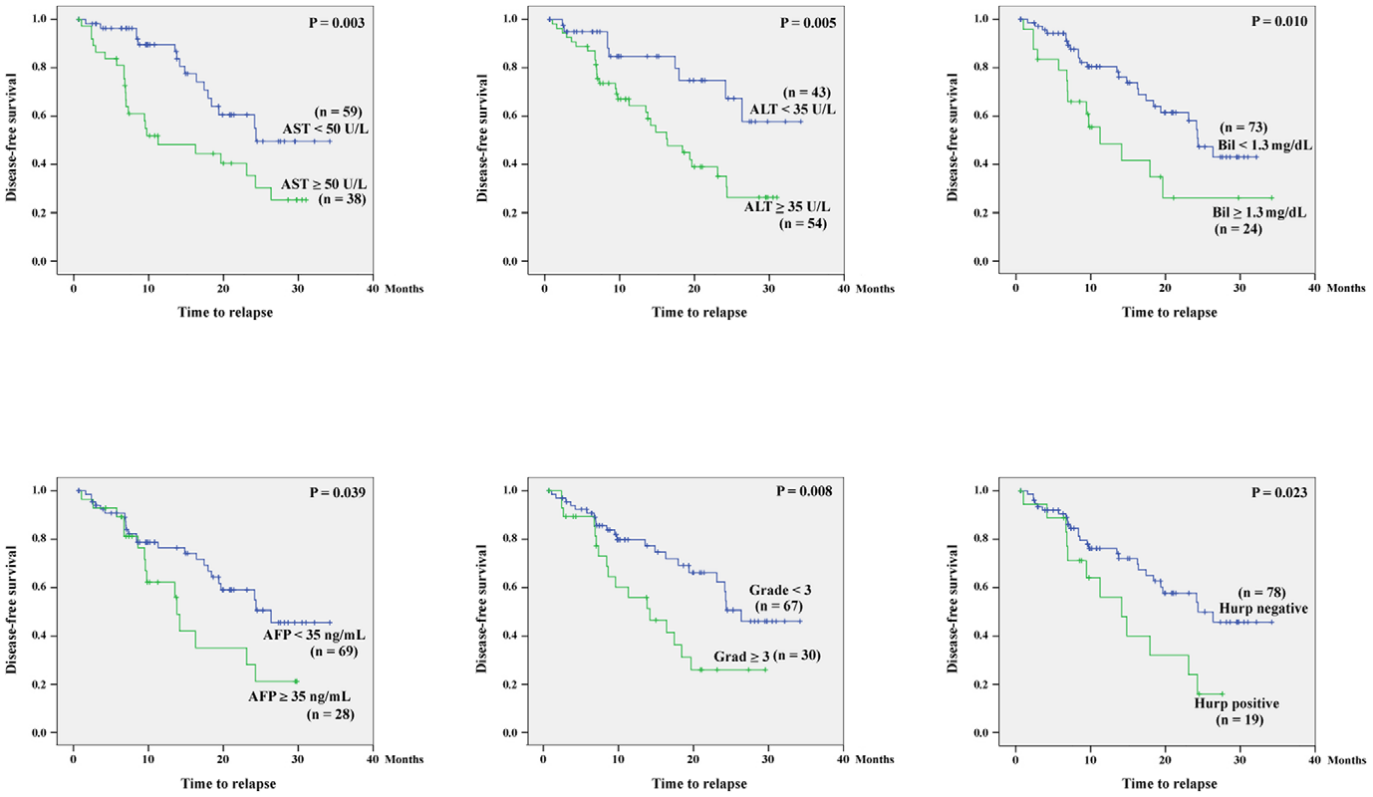
Comparison of the disease-free survivals between HCC patients with and without a statistically significant clinicopathological feature. n, number of HCC patients at risk.

The association between clinical parameters and disease-free survival is shown in table 2. Among the parameters, positive HURP expression, cytological grading ≥3, AFP ≥35 ng/mL, bilirubin ≥1.3 mg/dL, AST ≥50 U/L, and ALT ≥35 U/L were found to be associated with a shorter disease-free survival (Figure 2).

**Table 2:** Association between clinical parameters and disease-free survival.

Parameters	Category	No. of patients	Disease-free survival (months)		P (Log Rank)
			Mean	95% CI	
HURP expression	Negative	78	21.9	18.9–24.7	**0.023**
	Positive	19	14.1	10.2–18.1	
Treatment	RFA	79	20.2	17.4–23.1	0.770
	Surgical	18	19.4	14.7–24.3	
Sex	Female	35	21.1	16.7–25.4	0.772
	Male	62	19.4	16.5–22.3	
Age	<65 years	45	22.6	18.9–26.3	0.105
	≥65 years	52	17.1	14.2–20.0	
HBsAg	Negative	48	17.9	14.8–21.1	0.353
	Positive	49	21.5	17.9–25.0	
Anti-HCV	Negative	55	20.3	16.8–23.8	0.979
	Positive	42	18.9	15.6–22.2	
Alcoholism	No	78	20.8	17.8–23.8	0.606
	Yes	19	17.9	13.7–22.1	
Cirrhosis	No	10	19.4	15.2–23.6	0.910
	Yes	87	20.4	17.7–23.3	
Cytological grading	<3	67	22.6	19.7–25.7	**0.008**
	≥3	30	14.7	11.1–18.3	
Tumor number	Solitary	73	21.1	18.1–24.0	0.342
	>1	24	17.0	12.8–21.3	
Tumor size (diameter)	<3 cm	61	20.6	17.5–23.8	0.823
	≥3 cm	36	18.8	14.8–22.7	
Ascites	Absence	86	20.0	17.2–22.7	0.435
	Presence	11	21.9	16.3–27.4	
Alpha-fetoprotein	<35 ng/mL	69	22.0	19.0–25.1	**0.039**
	≥35 ng/mL	28	15.2	11.4–18.9	
Albumin	<4 g/dL	55	19.8	16.2–23.4	0.578
	≥4 g/dL	42	20.5	17.2–23.8	
Bilirubin	<1.3 mg/dL	73	21.3	18.7–23.9	**0.010**
	≥1.3 mg/dL	24	15.1	10.0–20.3	
Prothrombin time	<12 sec	35	19.8	16.5–23.2	0.902
	≥12 sec	62	20.8	17.3–24.2	
Creatinine	<1.0 mg/dL	48	21.7	18.1–25.4	0.391
	≥1.0 mg/dL	49	18.7	15.5–21.9	
AST	<50 U/L	59	23.5	20.3–26.8	**0.003**
	≥50 U/L	38	15.2	11.7–18.8	
ALT	<35 U/L	43	25.3	21.5–29.0	**0.005**
	≥35 U/L	54	16.5	13.7–19.4	

### Independent predictors of disease-free survival in the stepwise multivariate Cox proportional hazard model

**Figure 3:**
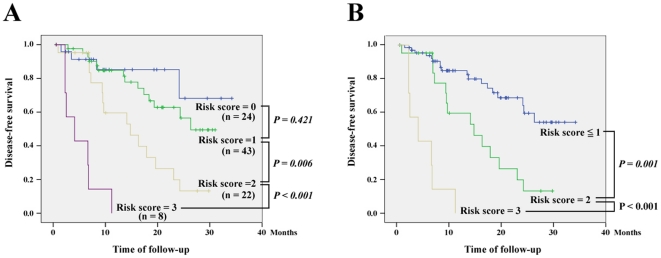
Comparison of the disease-free survivals among patients with various risk scores, which were defined as the number of independent predictors (positive HURP expression in HCC cells, AST ≥50 U/L, cytological grade ≥3, and tumor number >1) carried by each patient. (A) Comparison of the disease-free survivals among patients with various risk scores ranged from 0 to 3. (B) The patients with risk score = 0 and those with risk score = 1 were merged.

Using the stepwise multivariate Cox proportional hazard model, 4 factors remained as independent predictors for disease-free survival: positive HURP expression in HCC cells, AST ≥50 U/L, cytological grade ≥3, and tumor number >1. Their independence was also verified by bivariate correlation tests. It is worth noting that after adjusting for other confounding factors, the tumor number (which is not a significant parameter for disease-free survival in univariate analysis) became a significant factor in the Cox proportional hazard model. The hazard ratio (HR), 95% confidence interval (CI), and P values of the 4 independent predictors are listed in table 3. Finally, we assigned a risk score to each of the patients by calculating the number of independent predictors carried by each patient. The risk scores ranged from 0 to 3, with no patient carrying all 4 factors (figure 3). Because no significant difference was found in the disease-free survivals between patients with risk score = 0 and those with risk score = 1 (Figure 3A, *p* = 0.421), these two groups were merged (Figure 3B). The disease-free survivals were significantly different among the patients with risk scores ≤1, the patients with a risk score = 2, and the patients with a risk score = 3 (Figure 3B). In contrast, no difference (*p* = 0.91) was noted between the recurrence-free survivals of the 69 TNM stage I and 28 stage II patients.

**Table 3:** Independent predictors of disease-free survival in the stepwise multivariate Cox proportional hazard model.

Factors	HR	95%CI	P
HURP-positive in HCC cells	2.334	1.165–4.679	0.017
AST ≥50 U/L	3.697	1.868–7.319	<0.001
Cytological grade ≥3	4.249	2.061–8.759	<0.001
Tumor number >1	2.633	1.212–5.722	0.014

## Discussion

In general, hepatic resection was superior to RFA in HCCs eligible for surgical removal, particularly for tumors >3 cm [32]. When treating patients with solitary HCC ≤3 cm, RFA has a comparable recurrence free survival to surgical resection while being less invasive [33]. However, hepatic resection remains the treatment of choice for HCC in noncirrhotic patients because of the well-preserved hepatic function in the residual liver. On the other hand, RFA is safe and effective in managing HCC patients with liver cirrhosis, and its high repeatability makes it particularly valuable in controlling intrahepatic recurrences [34]. In two prospective randomized controlled trials comparing RFA with surgical resection, no significant difference was found in overall survival or recurrence-free survival. Further, lower complication rates were expectedly in patients treated with RFA [35], [36]. Therefore, the choice of therapy in very early stage HCC should depend on the patient's suitability for surgery, the performance status, the severity of liver cirrhosis, and the feasibility of RFA given the location of the tumor [37]. In our series, patients unsuitable for hepatectomy were subjected to RFA. Consistent with previous reports, the disease-free survival between these two methods was not significantly different (table 2). Thus, the bias of the different treatment methods should be negligible.

The heterogeneous nature of HCC has greatly hindered the search for effective molecular prognostic predictors. In a case-control study of 39 hepatitis C virus-related HCC cases (24 early stage) and 77 matched controls, neither des-gamma-carboxy prothrombin nor AFP was able to predict optimally the emergence of HCC [38]. Thus, even for HCC that has a homogeneous underlying disease, a reliable biomarker has yet to be found. According to the 6^th^ edition of AJCC/UICC TNM Classification, 69 and 28 BCLC stage A patients of the current study were classified as stage I, and II, respectively. However, those patients cannot be separated into prognosis distinguishable subgroups by using AJCC/UICC TNM staging system. In the present study, we demonstrated the independent prediction of disease-free survival in HCC by HURP expression in aspirated HCC cells. HURP is considered a stem cell marker and is undetectable in fully differentiated cells [39]. Similar to this observation, another stem cell marker, epithelial cell adhesion molecule (EpCAM), was found to be expressed dominantly in confluent multinodular type HCC and EpCAM expression levels predicted the recurrence of HCC [40]. Additionally, overexpression of Aurora B, a chromosomal passenger protein involved in chromosome segregation, spindle-checkpoint, and cytokinesis [41], independently predicted tumor invasion and poor prognosis of HCC [42]. The functional similarity between HURP and Aurora B further supports the predictive role of HURP in disease-free survival of HCC.

In recent years, tumor cell seeding along the needle tract has been found to be a risk associated with liver biopsy [43]. Fine needle aspiration cytology has proven to be a safe and accurate alternative for liver biopsy to identify the vast majority of HCC [44]. Therefore, HURP staining in aspirated HCC cells can potentially develop into a convenient method for predicting disease-free survival of HCC. However, there are some limitations associated with this technique. While HURP is named for its gene being up-regulated in human HCC, only 19.5% (19/97) of our HCC aspirated samples showed positive HURP expression. It is possible that in the remaining samples, the expression levels of HURP were too low for immunohistochemistry detection. Probably in these samples, the majority of HCC cells were in nonproliferating ‘out-of-cycle’ states. This caused the tumors to grow slowly, which resulted in a longer disease-free survival. Alternatively, in view of the assumption that HURP could be a stem cell marker, the low prevalence of HURP-positive cells in this study might reflect the fact that most of our HCCs arose from inflammation-related mutation induced by viral insults to hepatocytes (HBV or HCV infection in our series was over 90%), whereas HCCs that develop from *de novo* mutation of the naive hepatic stem cells only accounted for a minority of cases. In this study, we demonstrated that high AST, ALT, and bilirubin levels correlate with a shorter disease-free survival. This suggests that virus related hepatic necroinflammation plays an important role in HCC recurrence. At this time, it is not clear whether there is a pathway for the HCC cells that develop from virus related hepatocyte damage to evolve into HCCs with the signature of cancer stem cells. Finally, as mentioned in the introduction, redundant pathways that can compensate for HURP function have been proposed. As such, for HCCs that lack HURP expression, alternative oncogenic pathways unrelated to HURP over-expression are highly plausible.

Another puzzling aspect of the present data is that almost all HURP expression localized in the cytoplasm of the HCC cells. Importin-α1 was shown to be an independent predictor of early recurrence after HCC resection [45]. HURP is one of the spindle assembly factors whose activity is regulated by importins, and the steady-state distribution of HURP is determined by the continuous shuttling of HURP between the cytoplasm and nucleus via importin [46]. Most HURP studies have focused on its spindle assembly role in the nucleus during mitosis, while little is known regarding its function in the cytoplasm during interphase. Both HURP and Importin-α1 are over-expressed in HCCs with poor prognosis, which suggests important roles for these molecules in oncogenesis. The aberrant cytoplasmic over-expression of HURP in HCC might implicate an unexplored function in cell cycle regulation that demands further clarification.

Aside from HURP positivity, AST ≥50 U/L, cytological grade ≥3, and tumor number >1 were also found to be independent predictors for the disease-free survival in our HCC patients. Cytological grading represents the differentiation of the HCC cells and the tumor number may indicate a uni- or multi-focal tumor origin or alternatively, the staging of HCC. These factors are all suggestive of poor prognosis and have been documented by several studies [47], [48]. AST, ALT, bilirubin and AFP levels, which were identified to be significant prognosis predictors in univariate analysis, reflected either the degree of inflammation (AST, ALT and bilirubin) or the tumor burden (AFP). AFP also reflected the degree of hepatic inflammation in some cases [49]. In the literature, several lines of evidence indicate that hepatic inflammation is a prognostic predictor for HCC. The preoperative CRP level was shown to be associated with the aggressiveness of early recurrent HCC in a study of 124 patients who underwent hepatectomy [50]. In addition, studies regarding HCV-related HCC have shown that HCC almost always develops in a histologically abnormal liver and that the mere existence of chronic liver disease represents a potential risk for the development of HCC [51]. Indeed, chronic hepatic necroinflammation with its subsequent generation of reactive oxygen species can induce chromosomal mutations and eventually malignant transformation of proliferating hepatocytes. Likewise, poor liver function reserve, suggested by hyperbilirubinemia, was noted to be significantly associated with HCC occurrence in other studies [52]. A study enrolling 150 patients with a single HCC smaller than 5 cm in diameter treated by particle radiotherapy found that Child-Pugh classification was an independent risk factor for local recurrence [53]. Finally, in a retrospective study composed of 413 cirrhotic HCC patients receiving RFA and 648 cirrhotic HCC patients receiving surgical resection, serum AFP was found to be the only significant predictive factor for all survival analyses [54]. By combing the 4 independent predictors, which had been directly or indirectly associated with the prognosis of HCC in the literature, the risk scores of the patients with HCC separated the patients into three distinct groups with significantly different post-therapy prognoses. Therefore, in patients with BLCL stage A, risk scores that incorporate HURP staining are capable of providing further distinctions between different post-therapeutic prognosis groups. The small sample size of the present study, however, limited its clinical value. To validate the prognostic significance of HURP expression in a larger HCC cohort, a multi-center study would be extremely informative and should be conducted in the future.

To our knowledge, this is the first study regarding the clinical application of HURP expression in predicting the disease-free survival of HCC patients. A new risk score, composed of 4 independent predictors including HURP positivity in HCC cells, AST ≥50 U/L, cytological grade ≥3, and tumor number >1, separated HCC patients with BCLC stage A into three prognosis-distinguishable groups. These findings may be valuable in assessing the effects of therapeutic interventions for HCC patients with BCLC stage A, which is the most common stage discovered due to the early detection of HCC via orderly tumor survey. Finally, personalized therapy and follow-up for patients with early stage HCC can be pursued in the near future.
